# The Impact of Frailty on Outcomes of Proximal Aortic Aneurysm Surgery: A Nationwide Analysis

**DOI:** 10.3390/jcdd11010032

**Published:** 2024-01-20

**Authors:** Edward D. Percy, Thais Faggion Vinholo, Paige Newell, Supreet Singh, Sameer Hirji, Jake Awtry, Robert Semco, Muntasir Chowdhury, Alexander K. Reed, Sainath Asokan, Alexandra Malarczyk, Alexis Okoh, Morgan Harloff, Farhang Yazdchi, Tsuyoshi Kaneko, Ashraf A. Sabe

**Affiliations:** 1 Division of Cardiac Surgery, Department of Surgery, Brigham and Women’s Hospital, Harvard Medical School, Boston, MA 02115, USA; 2 Division of Cardiovascular Surgery, University of Pennsylvania Medical Center, Philadelphia, PA 19104, USA; 3 Department of Internal Medicine, Mount Sinai Hospital, New York, NY 10029, USA; 4 Department of Internal Medicine, Trinity Health System, Steubenville, OH 43952, USA; 5 Department of Cardiothoracic Surgery, Stanford University, Stanford, CA 94304, USA; 6 Department of Pediatrics, St. Christopher’s Hospital for Children, Philadelphia, PA 19134, USA; 7 Division of Cardiology, Emory University School of Medicine, Atlanta, GA 30322, USA; 8 Division of Cardiac Surgery, University of Michigan, Ann Arbor, MI 48109, USA; 9 Division of Cardiothoracic Surgery, Barnes-Jewish Hospital, Washington University in St Louis, St. Louis, MO 63110, USA

**Keywords:** aortic surgery, aortic aneurysm, frailty

## Abstract

(1) Background: This study examines frailty’s impact on proximal aortic surgery outcomes. (2) Methods: All patients with a thoracic aortic aneurysm who underwent aortic root, ascending aorta, or arch surgery from the 2016–2017 National Inpatient Sample were included. Frailty was defined by the Adjusted Clinical Groups Frailty Indicator. Outcomes of interest included in-hospital mortality and a composite of death, stroke, acute kidney injury (AKI), and major bleeding (MACE). (3) Results: Among 5745 patients, 405 (7.0%) met frailty criteria. Frail patients were older, with higher rates of chronic pulmonary disease, diabetes, and chronic kidney disease. There was no difference in in-hospital death (4.9% vs. 2.4%, *p* = 0.169); however, the frail group exhibited higher rates of stroke and AKI. Frail patients had a longer length of stay (17 vs. 8 days), and higher rates of non-home discharge (74.1% vs. 54.3%) than non-frail patients (both *p* < 0.001). Sensitivity analysis confirmed increased morbidity and mortality in frail individuals. After adjusting for patient comorbidities and hospital characteristics, frailty independently predicted MACE (OR 4.29 [1.88–9.78], *p* = 0.001), while age alone did not (OR 1.00 [0.99–1.02], *p* = 0.568). Urban teaching center status predicted a lower risk of MACE (OR 0.27 [0.08–0.94], *p* = 0.039). (4) Conclusions: Frailty is associated with increased morbidity in proximal aortic surgery and is a more significant predictor of mortality than age. Coordinated treatment in urban institutions may enhance outcomes for this high-risk group.

## 1. Introduction

Frailty is a multidimensional condition that involves the loss of function across several physiologic domains and predisposes patients to early physical decline and mortality [1,2,3]. It has been shown that frail patients who undergo surgery have worse outcomes following non-cardiac [4,5,6] and cardiac procedures [7,8,9]. Despite this, historically, there has not been a standard definition of what constitutes frailty [3], which has made it difficult to validate prior studies on frailty and systematically integrate this condition into surgical decision making. As a result, most preoperative risk assessment tools do not incorporate the effects of frailty in their evaluation [10]. The Society of Thoracic Surgeons Predicted Risk of Mortality score calculates risk scores specific to individual cardiac procedures but does not include frailty and has not been validated for aortic surgery. In the current era, however, frailty assessments such as the John Hopkins Adjusted Clinical Groups (ACGs) frailty indicator have become well validated [9,11,12,13,14], and it is essential to consider their implications in cardiac surgery.

A limited number of studies have assessed the impact of frailty in cardiac surgery, but they have demonstrated frail status to be associated with worse postoperative outcomes [7,8,9]. Proximal aortic surgery, in particular, carries significant morbidity and mortality risk due to the complexity of the procedure. Despite this, the impact of frailty on proximal aortic surgical outcomes is poorly understood and mainly derived from single-center studies [15,16,17]. As the age and medical complexity of patients who undergo proximal aortic surgery continues to increase, understanding the impact of frailty on surgical outcomes will become increasingly critical for perioperative assessment and informed surgical decision making. This study evaluates the impact of frailty on outcomes following aortic surgery for thoracic aortic aneurysms using a national representative database.

## 2. Materials and Methods

### 2.1. Database

The National Inpatient Sample (NIS) is the largest publicly available database in the United States, covering an estimated 20% of all inpatient hospitalizations annually across the country [18]. The NIS is a component of the Healthcare Cost and Utilization Project and is sponsored by the Agency for Healthcare Research and Quality (AHRQ). Weighted samples are used to represent over 95% of the United States’ population. The NIS is de-identified and considered a limited dataset; thus, the Mass General Brigham institutional review board determined this study to be exempt.

### 2.2. Study Population and Definitions

We retrospectively identified patients aged 18 years or older within the NIS database using International Classification of Diseases, Tenth Revision (ICD-10) diagnosis and procedure codes. All patients with a primary diagnosis of a thoracic aortic aneurysm who underwent proximal aorta surgery involving the aortic root, ascending aorta, or aortic arch between January 2016 and December 2017 were included in this study. Patients with thoracic aortic dissection and endocarditis were excluded by identifying and excluding corresponding ICD-10 codes. All patient characteristics, comorbidities, and outcomes were identified using relevant ICD-10 procedures and diagnosis codes. The Charlson Comorbidity Index, a validated prognostic tool that estimates the risk of mortality due to underlying comorbid diseases, was calculated for each patient [19]. Frailty was operationalized according to the Johns Hopkins ACGs frailty indicator [14]. The ACGs assess frailty using ten clusters of frailty-defining conditions (weight loss, frequent falls, malnutrition, incontinence, sacral ulcers, poor vision, gait, etc.) that have been well validated in prior studies [9,11,12,13]. This tool designates patients as “frail” if they have any of the frailty-defining conditions, and otherwise designates them “non-frail”.

Although the ACGs frailty indicator was the primary frailty definition used in the study, the impact of frailty was further validated in a sensitivity analysis using the Hospital Frailty Risk Score (HFS). This validated prognostic tool evaluates the risk of short-term mortality, readmission, and length of hospital stay given a patient’s degree of frailty, as defined by an established set of administrative codes [20,21]. Using the HFS, patients were assigned an overall frailty score for each admission that was further stratified into low (<5), intermediate (5–15), and high (>15) frailty risk according to previously validated cutoffs. Importantly, however, our analysis did not differentiate between different levels of frailty; patients with any degree of frailty were collectively classified as “frail”.

### 2.3. Study Outcomes

The primary endpoint was in-hospital all-cause mortality. Secondary endpoints included major adverse cardiac events (MACEs), stroke, bleeding, acute kidney injury (AKI), complete heart block, pacemaker insertion, length of stay (LOS), non-home discharge (to a skilled nursing facility or short-term hospital), and cost. MACE was defined as a composite of death, stroke, acute kidney injury, and major bleeding. Hospital costs were calculated by multiplying hospital specific cost-to-charge ratios by index charges.

### 2.4. Statistical Analysis

Continuous variables are presented as means and standard errors and were compared using independent t-tests. Categorical variables are presented as frequencies and their respective percentages and were compared using Rao–Scott $X^{2}$ tests. Appropriate survey procedures were used to generate weighted national estimates and variances that accounted for the clustering of outcomes within hospital sampling units and variation across regional strata, as recommended by the AHRQ [18,22]. Patient baseline characteristics and in-hospital outcomes were compared between frail and non-frail patients. The Charlson Comorbidity Index was calculated using ICD-10 diagnosis codes to assess the patients’ burden of medical comorbidities, adhering to a previously described methodology [23]. Multivariable regression models were used to determine the independent association of frailty with various postoperative outcomes, including LOS and cost. These models adjusted for patients characteristics and accounted for the sampling design and outcome clustering of the NIS using survey procedures. These models also adjusted for hospital location, teaching status, and hospital size (by number of patient beds), as identified within the NIS database. Analysis was conducted using SAS 9.4 (Cary, NC, USA). A *p*-value of ≤0.05 was the criterion for statistical significance.

## 3. Results

### 3.1. Patient Characteristics

Baseline demographics, comorbidities, frailty-defining conditions, and admission characteristics are presented in Table 1. A total of 5745 patients were included in the study period, with 405 (7.0%) of patients meeting the ACG criteria for frailty. Frail patients were significantly older (69 years vs. 62 years, *p* < 0.001) and more commonly female (40.7% vs. 30.7%, *p* = 0.046) compared to non-frail patients. Frail patients were more likely to have coronary artery disease (12.3% vs. 4.4%, *p* < 0.001), diabetes (9.9% vs. 4.4%, *p* = 0.031), chronic obstructive pulmonary disease (30.9% vs. 20.7%, *p* = 0.038), and chronic kidney disease (22.2% vs. 11.3%, *p* = 0.002), among other comorbidities. Frail patients had a significantly higher Charlson Comorbidity Index (*p* < 0.001) and were less likely to be admitted electively (*p* < 0.001).

### 3.2. Clinical Outcomes

There was no significant difference in in-hospital mortality between frail and non-frail patients (4.9% vs. 2.4%, *p* = 0.169). However, frail patients had a higher incidence of MACE (87.7% vs. 61.1%, *p* < 0.001), stroke (8.6% vs. 2.5%, *p* < 0.001), major bleeding (77.8% vs. 55.9%, *p* < 0.001), and AKI (33.3% vs. 15.4%, *p* < 0.001), as shown in Table 2. Frail patients also had a longer LOS compared to non-frail patients (17.0 days vs. 8.3 days, *p* < 0.001), a higher rate of non-home discharge (74.1% vs. 54.3%, *p* < 0.001), and higher total hospital charges ($419,515 vs. $228,573, *p* < 0.001). After adjusting for baseline characteristics and hospital factors, there was still no significant difference in in-hospital mortality, but frailty was independently predictive of MACE (odd ratio (OR) 4.29 [1.88–9.78], *p* = 0.001), bleeding (OR 2.63 [1.47–4.67], *p* = 0.001), and AKI (OR 2.09 [1.13–3.84], *p* = 0.018) (Table 3).

An additional adjusted analysis in the same population examined the effect of age on patient outcomes. Age alone was predictive of AKI (OR 1.02 [1.01–1.04], *p* = 0.013), but was not predictive of MACE (OR 1.00 [0.99–1.02], *p* = 0.568), in-hospital mortality (OR 1.02 [0.96–1.08], *p* = 0.514), or major bleeding (OR 1.00 [0.98–1.02], *p* = 0.940) (Supplemental Table S1).

### 3.3. Multivariable Analysis in Frail Patients

**Figure 1:**
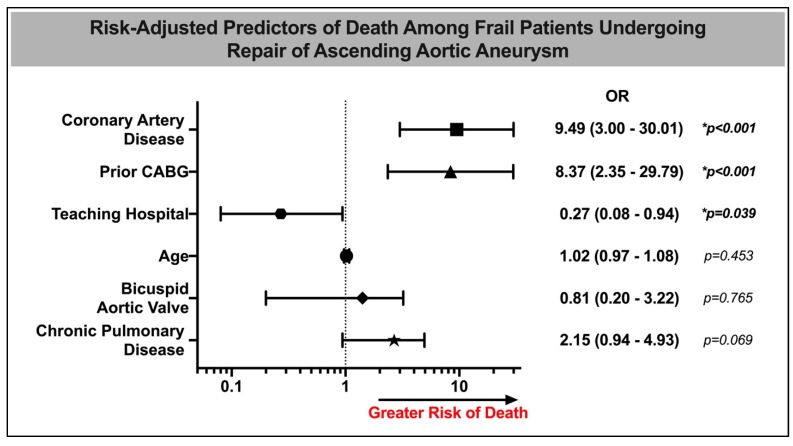
Risk-adjusted predictors of death among frail patients undergoing repair of ascending aortic aneurysm. CABG—coronary artery bypass grafting, OR—odds ratio. * *p*-value ≤ 0.05 was considered statistically significant.

A multivariable analysis of in-hospital mortality for frail patients was conducted, as shown in Figure 1. Among frail patients, the presence of coronary artery disease (OR 9.49 [3.00–30.09], *p* < 0.001) and prior coronary bypass (OR 8.37 [2.35–29.79], *p* < 0.001) were independent predictors of in-hospital mortality. In contrast, hospital status as an urban teaching center was associated with decreased in-hospital mortality (OR 0.27 [0.08–0.94], *p* = 0.039) (Table 4).

### 3.4. Sensitivity Analysis

A sensitivity analysis was performed to confirm the association of frailty with clinical outcomes in this cohort. In total, 1149 patients registered on the HFS risk scoring system. Overall, 772 (67.2%) were low-risk, 366 (31.9%) were intermediate-risk, and 11 (0.9%) were considered high-risk. In-hospital mortality was significantly higher in the high-risk group compared to intermediate- and low-risk groups, respectively (9.1% vs. 5.7% vs. 1.0%, *p* < 0.001) (Supplemental Table S2). The risk of stroke and renal failure was also increased with incremental increases in frailty risk.

## 4. Discussion

Our study provides the first evaluation of the association of frailty with outcomes of proximal aortic surgery on a national scale, revealing several noteworthy findings. Foremost, frail patients were older with more medical comorbidities and had increased complication rates, longer hospitalizations, increased likelihood of discharge to a location other than home, and increased cost. Notably, frailty was not associated with increased in-hospital mortality. This relationship was confirmed through a sensitivity analysis utilizing an additional frailty scoring system. Of patients who were frail, age alone was not predictive of in-hospital mortality or MACE, though prior coronary artery bypass grafting (CABG) and coronary artery disease were independent predictors of in-hospital mortality. Finally, management at an urban academic medical center was protective for frail patients undergoing proximal aortic surgery. These findings demonstrate that frailty is associated with significantly increased morbidity in proximal aortic surgery and is a more significant predictor of mortality than age alone. Efforts to coordinate the treatment of frail patients in urban teaching hospitals may improve outcomes in this high-risk group.

This study demonstrated that frail patients have higher unadjusted risk of MACE, postoperative AKI, bleeding, and stroke. Importantly, after adjusting for patient and hospital-level factors, frailty continued to be associated with higher rates of MACE, AKI, and bleeding. The multifactorial nature of frailty can likely explain this increased risk of post-procedural complications. First, frail patients have increased disease burden with poor physiological reserve, placing them at risk for developing postoperative complications [7,8,9,13]. Frail patients have been shown to have elevated inflammatory markers, including C-reactive protein, IL-6, and TNF-α, and elevated levels of Factor VIII and D-dimer, which raise concern for an underlying coagulopathic state which may predispose them to stroke and myocardial infarction [24,25]. Moreover, this population is prone to vascular fragility, which may account for the increased risk of MACE and bleeding [24,26]. Underlying disease states that are more prevalent in frail patients, such as malnutrition, have been shown to increase rates of AKI [27], which age alone does not capture. This is supported by our study demonstrating that age alone is not as predictive of poor postoperative outcomes. As the United States population continues to age, greater numbers of older patients are undergoing proximal aortic surgery [15,28]. Historically, advanced age and frailty have been used synonymously due to their high co-occurrence rate [3]. However, this work demonstrates that advanced age and frailty are fundamentally distinct and reinforces recent findings that age alone is not a reliable predictor of surgical outcome [29]. As frailty accounts for age, comorbidities, and a patient’s overall physical condition, it is a more comprehensive measure of overall health and better accounts for non-disease related factors that can result in post-procedural complications.

Our study’s finding that frail patients have a greater comorbidity burden and worse perioperative outcomes is consistent with prior studies [7,8,9,13]. Although this study did not demonstrate a significant difference in mortality, most frail patients are deemed non-surgical candidates and may not even be offered surgical treatment; the similar mortality rates between our frail and non-frail patients may be attributable to this selection bias. Furthermore, the NIS only permits study of inpatient mortality, while several studies have demonstrated that frailty is associated with increased long-term mortality [6,13,17]. Complicating the integration of our study with existing literature is the variation in how frailty has been defined in previous studies, which limits the direct comparison of results. Ganapathi et al. found that frailty was associated with increased mortality, but in their novel definition of frailty they incorporated psoas muscle volume and presence of anemia [17]. While both the ACG frailty indicator used in our study and the approach used by Ganapathi et al. assigned frailty status using similar factors including protein malnutrition and weight loss, differing definitions of frailty highlight the need for a comprehensive and consistent frailty definition to assess the true relationship between frailty and cardiac surgery outcomes. We suggest that the ACG frailty system assessment that was used in this study and has been previously validated may deserve further study and use in the cardiac surgery population.

This study demonstrated that coronary artery disease and prior CABG were shown to be independent predictors of in-hospital mortality for frail patients undergoing proximal aortic surgery. Given their diminished physiologic reserve, frail patients with underlying coronary artery disease are more prone to post-operative MACE due to an impaired ability to compensate for the myocardial stunning observed after the use of cardiopulmonary bypass [30]. For patients with prior CABG, the effect of repeat sternotomy increases the level of technical complexity and risk of complications, and increased risk of reoperation in ischemic disease further predisposes frail patients to post-operative complications [31]. For these reasons, frail patients with a history of coronary artery disease or repeat CABG should be carefully evaluated with a low threshold to refer to a higher volume center if appropriate.

The relationship between cardiac surgical volume and improved perioperative outcomes has been well characterized [32,33,34,35]. It has been previously shown that institutions that perform at least 20–25 proximal aortic surgeries annually have lower perioperative mortality rates [35]. This association supports our findings that treatment at urban academic medical centers, which are typically high in volume, is protective against mortality for frail patients. Frailty is a multifactorial condition that is best managed with a team-based, multidisciplinary, postoperative treatment plan [36]. Large urban centers with higher surgical volumes are more likely to have the resources necessary to provide this kind of high-quality care to frail patients, and transfer of high-risk frail patients to these centers should be carefully considered.

In addition to its impact on operative outcomes, the diminished physiologic reserve in frail patients is reflected in their longer post-operative hospital stay, greater rate of non-home discharge, and higher hospital costs compared to non-frail patients. Improving the multidisciplinary care of frail patients has the potential to improve outcomes following proximal aortic surgery and improve the cost-effectiveness of post-procedural care. Including a frailty assessment in the preoperative evaluation of these patients will help to appropriately risk-stratify and identify those at highest risk for morbidity and mortality. Implementation of preoperative optimization protocols for nutrition, polypharmacy, and comorbidity management as well as customized postoperative pathways may improve the surgical outcomes and overall care of this high-risk group. The care of this population has important economic implications for healthcare systems and policy makers, as systematic improvement in the multidisciplinary patient care of patients with frailty may result in financial savings.

Based on our findings, we propose considering the integration of frailty testing into routine preoperative workups for patients undergoing proximal aortic surgery. Using a specific indicator, such as the ACG frailty system assessment, could provide valuable insights for tailored patient care, enhance risk stratification, and contribute to data-driven clinical decision making.

### Limitations

As this study utilized NIS, it is subjected to the inherent limitation of large, administrative databases. This study is susceptible to missing or inaccurate coding across healthcare facilities. Additionally, the NIS database dose not capture granular information such as medication, laboratory data, imaging results, and procedural characteristics that have the potential to skew our results. To increase the rigor of this study, we validated the functionality of this administrative database frailty score to be more rigorous, but application of frailty testing to real world aortic patients may be more easily accomplished using other, more clinically oriented scales. Finally, NIS only captures inpatient outcomes. Further research on intermediate and long-term outcomes of frail patients undergoing aortic surgery is necessary to characterize this relationship more completely. Despite efforts to ensure data reliability, the study’s conclusion should be interpreted cautiously, recognizing the constraints imposed by utilizing nationwide data and hospital codes.

Our study assigned an overall frailty score (low, intermediate, high), yet this aspect was not fully considered in the statistical analyses. Recognizing the impact of varying degrees of frailty on outcomes, a stratified analysis would have more comprehensively elucidated the nuanced relationship between frailty severity and surgical outcomes.

Lastly, our patient cohort includes proximal aortic surgery of varying degrees of complexity without making distinctions thereof within the analysis. This may have hindered potential insight into the relationship between surgical complexity and outcomes in frailty patients.

## 5. Conclusions

Frailty is a complex condition in which diminished physiologic reserve places patients at increased risk of post-operative complications. In this study, we present the first national assessment of the relationship between patient frailty and proximal aortic surgery outcomes. Frail status was significantly associated with worse post-operative morbidity, particularly in those with a history of coronary artery disease. Importantly, among patients with frailty, age alone was not an independent predictor of postoperative outcomes. With an aging population, emphasizing frailty to be a more accurate predictor of postoperative outcomes may allow proximal aortic surgery to be offered to more elderly but otherwise non-frail patients with improved outcomes. With the increased rate of post-procedural complications, longer hospital stays, and increased hospitalization costs, improving the identification and multidisciplinary care of frail patients undergoing proximal aortic surgery is critical for patients, physicians, and health policy makers.

## Figures and Tables

**Table 1:** Baseline patient characteristics.

	Non-Frail (n = 5340)	Frail (n = 405)	*p*-Value
*Demographics*			
Mean Age, years (SD)	61.8 (0.4)	68.7 (1.3)	<0.001 *
Female [N (%)]	1640 (30.7%)	165 (40.7%)	<0.05 *
*Comorbidities [N (%)]*			
Atrial Fibrillation	1295 (24.3%)	115 (28.4%)	0.40
Coronary Artery Disease	235 (4.4%)	50 (12.3%)	<0.001 *
Dyslipidemia	2430 (45.5%)	180 (44.4%)	0.85
Prior Myocardial Infarction	240 (4.5%)	40 (9.9%)	0.03 *
Congestive Heart Failure	1255 (23.5%)	100 (24.7%)	0.80
Chronic Pulmonary Disease	1105 (20.7%)	125 (30.9%)	0.04 *
Diabetes	235 (4.4%)	40 (9.9%)	0.03 *
Hypertension	3635 (68.1%)	250 (61.7%)	0.24
Bicuspid Aortic Valve	1285 (24.1%)	45 (11.1%)	<0.01 *
Chronic Kidney Disease	605 (11.3%)	90 (22.2%)	<0.01 *
Prior CABG	90 (1.7%)	10 (2.5%)	0.60
Number of Charlson Comorbidities [N (%)]			
*One*	2360 (44.2%)	75 (18.5%)	<0.001 *
*Two*	1745 (32.7%)	120 (29.6%)	
*Three or more*	1235 (23.1%)	210 (51.9%)	
*Frailty-Defining Conditions [N (%)]*			
Malnutrition	0 (0%)	95 (23.5%)	
Dementia	0 (0%)	260 (64.2%)	
Impaired Vision	0 (0%)	0 (0%)	
Sacral Ulcer	0 (0%)	20 (4.9%)	
Urine Incontinence	0 (0%)	0 (0%)	
Fecal Incontinence	0 (0%)	0 (0%)	
Weight Loss	0 (0%)	10 (2.5%)	
Lacking Social Support	0 (0%)	20 (4.9%)	
Difficulty Walking	0 (0%)	25 (6.2%)	
Mechanical Fall	0 (0%)	10 (2.5%)	
*Admission Characteristics*			
Admission on Weekend	145 (2.7%)	35 (8.6%)	<0.01 *
Elective Admission	4425 (83.2%)	275 (67.9%)	<0.01 *
*Transfer Status*			
Not Transferred	5060 (94.9%)	360 (88.9%)	0.07
Transferred From a Different Acute Care Hospital	230 (4.3%)	35 (8.6%)	
Transferred From Another Type of Health Facility	40 (0.8%)	10 (2.5%)	

CABG—coronary artery bypass grafting, SD—standard deviation. * *p*-value ≤ 0.05 was considered statistically significant. Boldface values denote statistical significance.

**Table 2:** Observed outcomes and hospital factors of proximal aortic surgery for frail and non-frail patients.

	Non-Frail (n = 5340)	Frail (n = 405)	*p*-Value
*In-Hospital Outcomes*			
MACE [N (%)]	3265 (61.1%)	355 (87.7%)	<0.01 *
Acute Kidney Injury [N (%)]	825 (15.4%)	135 (33.3%)	<0.01 *
Complete Heart Block [N (%)]	330 (6.2%)	45 (11.1%)	0.07
Major Bleed [N (%)]	2985 (55.9%)	315 (77.8%)	<0.01 *
Stroke [N (%)]	135 (2.5%)	35 (8.6%)	<0.01 *
Death [N (%)]	130 (2.4%)	20 (4.9%)	0.17
Pacemaker Insertion [N (%)]	130 (2.4%)	30 (7.4%)	<0.01 *
Non-home Discharge [N (%)]	2895 (54.3%)	300 (74.1%)	<0.01 *
LOS (Mean Days, SE)	8.3 (0.2)	17.0 (1.7)	<0.01 *
Cost (Mean USD $, SE)	228,573 (8855)	419,515 (46,771)	<0.01 *
*Hospital Factors*			
Bed Size [N (%)]			0.78
*Small*	405 (7.6%)	30 (7.4%)	
*Medium*	835 (15.6%)	75 (18.5%)	
*Large*	4100 (76.8%)	300 (74.1%)	
Ownership of Hospital [N (%)]			0.77
*Government, Nonfederal*	410 (7.7%)	30 (7.4%)	
*Private, Not-for-Profit*	4630 (86.7%)	345 (85.2%)	
*Private, Invest Own*	300 (5.6%)	30 (7.4%)	
Teaching Status of Hospital [N (%)]			--
*Rural*	65 (1.2%)	0 (0%)	
*Urban Non-Teaching*	395 (7.4%)	10 (2.5%)	
*Urban Teaching*	4880 (91.4%)	395 (97.5%)	
Region of Hospital [N (%)]			0.82
*Northeast*	1210 (22.7%)	100 (24.7%)	
*Midwest*	1375 (25.7%)	105 (25.9%)	
*South*	1725 (32.3%)	140 (34.6%)	
*West*	1030 (19.3%)	60 (14.8%)	

LOS—length of stay; MACE—major adverse cardiac event defined as a composite of death, stroke, acute kidney injury, and major bleeding; SE—standard error. * *p*-value ≤ 0.05 was considered statistically significant. Boldface values denote statistical significance.

**Table 3:** Adjusted analysis showing frailty as a predictor for various post-operative outcomes.

Outcome	Odds Ratio	95% Confidence Interval		*p*-Value
Death	1.40	0.43	4.50	0.575
Stroke	1.61	0.11	23.75	0.727
Acute Kidney Injury	2.09	1.13	3.84	0.018 *
Pacemaker Insertion	3.05	0.96	9.72	0.060
Complete Heart Block	1.55	0.65	3.71	0.325
Major Bleeding	2.63	1.47	4.67	0.001 *
MACE	4.29	1.88	9.78	0.001 *

MACE—major adverse cardiac event defined as a composite of death, stroke, acute kidney injury, and major bleeding. * *p*-value ≤ 0.05 was considered statistically significant. Boldface values denote statistical significance.

**Table 4:** Predictors of in-hospital mortality among frail patients undergoing proximal aortic surgery.

Variable	Odds Ratio	95% Confidence Interval		*p*-Value
Age	1.02	0.97	1.08	0.45
Female	0.47	0.18	1.26	0.13
Dyslipidemia	0.20	0.07	0.62	0.005 *
Bicuspid Aortic Valve	0.81	0.20	3.22	0.75
Hypertension	0.60	0.18	2.04	0.41
Chronic Pulmonary Disease	2.15	0.94	4.93	0.07
Chronic Kidney Disease	1.95	0.43	8.77	0.38
Coronary Artery Disease	9.49	3.00	30.01	<0.001 *
Atrial Fibrillation	0.80	0.29	2.20	0.66
Congestive Heart Failure	2.15	0.96	4.85	0.064
Teaching Hospital	0.27	0.08	0.94	0.039 *
Prior CABG	8.37	2.35	29.79	0.001 *

CABG—coronary artery bypass grafting. * *p*-value ≤ 0.05 was considered statistically significant. Boldface values denote statistical significance.

## Data Availability

The data used in this study are from the National Inpatient Sample (NIS) repository, which is a publicly available dataset developed by the Healthcare Cost and Utilization Project (HCUP) sponsored by the Agency for Healthcare Research and Quality (AHRQ). Researchers interested in accessing the data can obtain them from the AHRQ following their data use agreement and access procedures. Information on how to access the NIS data is available on the AHRQ website (http://www.hcup-us.ahrq.gov/, accessed on 19 January 2024).

## Supplementary Materials

The following supporting information can be downloaded at: https://www.mdpi.com/article/10.3390/jcdd11010032/s1, Table S1: Adjusted analysis showing age as a predictor for various post-operative outcomes; Table S2: Sensitivity analysis examining in-hospital outcomes stratified by hospital frailty risk score.

## References

[1] Xue Q.-L. (2011). The frailty syndrome: Definition and natural history. Clin. Geriatr. Med..

[2] Fried L.P., Tangen C.M., Walston J., Newman A.B., Hirsch C., Gottdiener J., Seeman T., Tracy R., Kop W.J., Burke G. (2001). Frailty in older adults: Evidence for a phenotype. J. Gerontol. A Biol. Sci. Med. Sci..

[3] Kojima G., Liljas A.E.M., Iliffe S. (2019). Frailty syndrome: Implications and challenges for health care policy. Risk Manag. Healthc. Policy.

[4] Morley J.E., Vellas B., van Kan G.A., Anker S.D., Bauer J.M., Bernabei R., Cesari M., Chumlea W., Doehner W., Evans J. (2013). Frailty consensus: A call to action. J. Am. Med. Dir. Assoc..

[5] Shinall M.C., Youk A., Massarweh N.N., Shireman P.K., Arya S., George E.L., Hall D.E. (2020). Association of preoperative frailty and operative stress with mortality after elective vs. emergency surgery. JAMA Netw. Open.

[6] Oakland K., Nadler R., Cresswell L., Jackson D., Coughlin P.A. (2016). Systematic review and meta-analysis of the association between frailty and outcome in surgical patients. Ann. R. Coll. Surg. Engl..

[7] Sepehri A., Beggs T., Hassan A., Rigatto C., Shaw-Daigle C., Tangri N., Arora R.C. (2014). The impact of frailty on outcomes after cardiac surgery: A systematic review. J. Thorac. Cardiovasc. Surg..

[8] Lee D.H., Buth K.J., Martin B.-J., Yip A.M., Hirsch G.M. (2010). Frail patients are at increased risk for mortality and prolonged institutional care after cardiac surgery. Circulation.

[9] Iyengar A., Goel N., Kelly J.J., Han J., Brown C.R., Khurshan F., Chen Z., Desai N. (2020). Effects of frailty on outcomes and 30-day readmissions after surgical mitral valve replacement. Ann. Thorac. Surg..

[10] Bagnall N.M., Faiz O., Darzi A., Athanasiou T. (2013). What is the utility of preoperative frailty assessment for risk stratification in cardiac surgery?. Interact. Cardiovasc. Thorac. Surg..

[11] Neuman H.B., Weiss J.M., Leverson G., O’Connor E.S., Greenblatt D.Y., Loconte N.K., Greenberg C.C., Smith M.A. (2013). Predictors of short-term postoperative survival after elective colectomy in colon cancer patients ≥ 80 years of age. Ann. Surg. Oncol..

[12] McIsaac D.I., Bryson G.L., van Walraven C. (2016). Association of frailty and 1-year postoperative mortality following major elective noncardiac surgery: A population-based cohort study. JAMA Surg..

[13] Tran D.T.T., Tu J.V., Dupuis J.-Y., Bader Eddeen A., Sun L.Y. (2018). Association of frailty and long-term survival in patients undergoing coronary artery bypass grafting. J. Am. Heart Assoc..

[14] Abrams C., Roy L., Weiner J.P. (2003). Development and Evaluation of the Johns Hopkins University Risk Adjustment Models for Medicare+Choice Plan Payment.

[15] Williams J.B., Peterson E.D., Zhao Y., O’Brien S.M., Andersen N.D., Miller D.C., Chen E.P., Hughes G.C. (2012). Contemporary results for proximal aortic replacement in North America. J. Am. Coll. Cardiol..

[16] Krähenbühl E.S., Immer F.F., Stalder M., Englberger L., Eckstein F.S., Schmidli J., Carrel T.P. (2008). Technical advances improved outcome in patients undergoing surgery of the ascending aorta and/or aortic arch: Ten years experience. Eur. J. Cardiothorac. Surg..

[17] Ganapathi A.M., Englum B.R., Hanna J.M., Schechter M.A., Gaca J.G., Hurwitz L.M., Hughes G.C. (2014). Frailty and risk in proximal aortic surgery. J. Thorac. Cardiovasc. Surg..

[18] HCUP-US NIS Overview. https://hcup-us.ahrq.gov/nisoverview.jsp.

[19] Charlson M.E., Pompei P., Ales K.L., MacKenzie C.R. (1987). A new method of classifying prognostic comorbidity in longitudinal studies: Development and validation. J. Chronic Dis..

[20] Eckart A., Hauser S.I., Haubitz S., Struja T., Kutz A., Koch D., Neeser O., Meier M.A., Mueller B., Schuetz P. (2019). Validation of the hospital frailty risk score in a tertiary care hospital in Switzerland: Results of a prospective, observational study. BMJ Open.

[21] Gilbert T., Neuburger J., Kraindler J., Keeble E., Smith P., Ariti C., Arora S., Street A., Parker S., Roberts H.C. (2018). Development and validation of a Hospital Frailty Risk Score focusing on older people in acute care settings using electronic hospital records: An observational study. Lancet.

[22] Khera R., Angraal S., Couch T., Welsh J.W., Nallamothu B.K., Girotra S., Chan P.S., Krumholz H.M. (2017). Adherence to methodological standards in research using the National Inpatient Sample. JAMA.

[23] Quan H., Li B., Couris C.M., Fushimi K., Graham P., Hider P., Januel J.-M., Sundararajan V. (2011). Updating and validating the Charlson comorbidity index and score for risk adjustment in hospital discharge abstracts using data from 6 countries. Am. J. Epidemiol..

[24] Walston J., McBurnie M.A., Newman A., Tracy R.P., Kop W.J., Hirsch C.H., Gottdiener J., Fried L.P. (2002). Frailty and activation of the inflammation and coagulation systems with and without clinical comorbidities: Results from the Cardiovascular Health Study. Arch. Intern. Med..

[25] Langmann G.A., Perera S., Ferchak M.A., Nace D.A., Resnick N.M., Greenspan S.L. (2017). Inflammatory markers and frailty in long-term care residents. J. Am. Geriatr. Soc..

[26] Dodson J.A., Hochman J.S., Roe M.T., Chen A.Y., Chaudhry S.I., Katz S., Zhong H., Radford M.J., Udell J.A., Bagai A. (2018). The association of frailty with in-hospital bleeding among older adults with acute myocardial infarction: Insights from the ACTION Registry. JACC Cardiovasc. Interv..

[27] Li C., Xu L., Guan C., Zhao L., Luo C., Zhou B., Zhang X., Wang J., Zhao J., Huang J. (2020). Malnutrition screening and acute kidney injury in hospitalised patients: A retrospective study over a 5-year period from China. Br. J. Nutr..

[28] Tkatch R., Musich S., MacLeod S., Alsgaard K., Hawkins K., Yeh C.S. (2016). Population Health Management for Older Adults: Review of Interventions for Promoting Successful Aging Across the Health Continuum. Gerontol. Geriatr. Med..

[29] Wanamaker K.M., Hirji S.A., Del Val F.R., Yammine M., Lee J., McGurk S., Shekar P., Kaneko T. (2019). Proximal aortic surgery in the elderly population: Is advanced age a contraindication for surgery?. J. Thorac. Cardiovasc. Surg..

[30] Anselmi A., Abbate A., Girola F., Nasso G., Biondi-Zoccai G.G.L., Possati G., Gaudino M. (2004). Myocardial ischemia, stunning, inflammation, and apoptosis during cardiac surgery: A review of evidence. Eur. J. Cardiothorac. Surg..

[31] Park C.B., Suri R.M., Burkhart H.M., Greason K.L., Dearani J.A., Schaff H.V., Sundt T.M. (2010). Identifying patients at particular risk of injury during repeat sternotomy: Analysis of 2555 cardiac reoperations. J. Thorac. Cardiovasc. Surg..

[32] Chikwe J., Toyoda N., Anyanwu A.C., Itagaki S., Egorova N.N., Boateng P., El-Eshmawi A., Adams D.H. Relation of Mitral Valve Surgery Volume to Repair Rate, Durability, and Survival. J. Am. Coll. Cardiol..

[33] Shuhaiber J., Isaacs A.J., Sedrakyan A. (2015). The Effect of Center Volume on In-Hospital Mortality After Aortic and Mitral Valve Surgical Procedures: A Population-Based Study. Ann. Thorac. Surg..

[34] Lin H.-C., Xirasagar S., Tsao N.-W., Hwang Y.-T., Kuo N.-W., Lee H.-C. (2008). Volume-outcome relationships in coronary artery bypass graft surgery patients: 5-year major cardiovascular event outcomes. J. Thorac. Cardiovasc. Surg..

[35] Mori M., Shioda K., Wang X., Mangi A.A., Yun J.J., Darr U., Elefteriades J.A., Geirsson A. (2018). Perioperative Risk Profiles and Volume-Outcome Relationships in Proximal Thoracic Aortic Surgery. Ann. Thorac. Surg..

[36] Robinson T.N., Walston J.D., Brummel N.E., Deiner S., Brown C.H., Kennedy M., Hurria A. (2015). Frailty for Surgeons: Review of a National Institute on Aging Conference on Frailty for Specialists. J. Am. Coll. Surg..

