# Supplementary material for: The Impact of Frailty on Outcomes of Proximal Aortic Aneurysm Surgery: A Nationwide Analysis
Source: J Cardiovasc Dev Dis. 2024 Jan 20;11(1):32. doi: 10.3390/jcdd11010032 (PMC10816774; doi:10.3390/jcdd11010032)
Supplement: Supplementary file 1 [file jcdd-11-00032-s001.zip › jcdd-2808180-supplementary.pdf]

Supplementary Materials:

Table S1. Adjusted Analysis Showing Age As A Predictor For Various Post-Operative Outcomes.

Outcome	Odds Ratio	95% Confidence Interval		P-Value
**Death**	1.02	0.96	1.08	0.514
**Stroke**	1.12	0.99	1.27	0.084
**Acute Kidney Injury**	1.02	1.01	1.04	**0.013***
**Pacemaker Insertion**	0.99	0.95	1.03	0.699
**Complete Heart Block**	1.00	0.97	1.03	0.894
**Major Bleeding**	1.00	0.98	1.02	0.940
**MACE**	1.00	0.99	1.02	0.568

MACE- Major Adverse Cardiac Event defined as composite of death, stroke, acute kidney injury, and major bleeding. *P-value ≤ 0.05 was considered statistically significant.

Table S2. Sensitivity Analysis Examining In-Hospital Outcomes Stratified by Hospital Frailty Risk Score.

	Overall (N=1149)	Low HFS (N=772)	Intermediate HFS (N=366)	High HFS (N=11)	p-value
**In-Hospital Outcomes**					
**Mortality**	30 (2.6%)	8 (1.0%)	21 (5.7%)	1 (9.1%)	**<0.001**
**Stroke**	34 (3.0%)	4 (0.5%)	24 (6.6%)	6 (54.6%)	**<0.001**
**Renal failure**	192 (16.7%)	58 (7.5%)	130 (35.5%)	4 (36.4%)	**<0.001**
**Complete heart block**	75 (6.5%)	48 (6.2%)	26 (7.1%)	1 (9.1%)	0.80
**Major bleeding**	660 (57.4%)	403 (52.2%)	252 (68.9%)	5 (45.5%)	**<0.001**

HFS = Hospital Frailty Risk Score (Low: <5, Intermediate: 5-15, High: >15).
Boldface values denote statistical significance. Categorical variables were analyzed using chi-square tests.
